# Prevalence of Contributing Factors Leading to the Development of Insulin Resistance Among Male Medical Students at a Private College in Saudi Arabia

**DOI:** 10.7759/cureus.48269

**Published:** 2023-11-04

**Authors:** Ibrahim K Alghamdi, Abdullah M Alrefai, Theyab A Alghamdi, Amro T Nawawi, Yousria A Badawy

**Affiliations:** 1 Medicine, Ibn Sina National College for Medical Studies, Jeddah, SAU; 2 Family Medicine, Ibn Sina National College for Medical Studies, Jeddah, SAU; 3 Family Medicine, Alexandria University, Alexandria, EGY

**Keywords:** male medical students, insulin resistance, body mass index, lifestyle habits, obesity, systolic blood pressure, diastolic blood pressure, daily physical activity, day sleep, risk factors

## Abstract

Background

Insulin resistance can result from various genetic and lifestyle factors. Initially, symptoms of insulin resistance may not be readily noticeable, but as the condition progresses, individuals may start experiencing symptoms. This study aimed to investigate the factors contributing to the development of insulin resistance among medical students at a private college in Saudi Arabia.

Methodology

We conducted a cross-sectional study using a convenient non-probability sampling technique, with a sample size of 241 participants. We employed validated questionnaires to gather information on physical activity, sleep, dietary habits, and stress. Specifically, we used the International Physical Activity Questionnaire (IPAQ)-Short Form for assessing physical activity, the Single-Item Sleep Quality Scale (SQS) for evaluating sleep quality, the Healthy Eating Quiz (HEQ) to gauge dietary patterns, and the stress questionnaire designed by the International Stress Management Association (ISMA) to measure stress levels. Additionally, we collected anthropometric measurements, as well as systolic and diastolic blood pressure readings. We calculated prevalence using percentages and employed the chi-square test to analyze variables, with a significance level set at p-values <0.05.

Results

This study investigated risk factors associated with lifestyle, focusing on waist circumference as an indicator of insulin resistance. Our findings revealed that a majority of individuals with high waist circumference were physically inactive and more susceptible to stress, and this difference was statistically significant when compared to those with normal waist circumference. Additionally, we observed that sleep deprivation and poor nutrition were more prevalent among individuals with high waist circumference, although these differences were not statistically significant.

Conclusions

This study highlights a high prevalence of elevated waist circumference, indicating insulin resistance, among medical students. Furthermore, it underscores the significant presence of well-known risk factors associated with insulin resistance within this population of medical students.

## Introduction

Insulin resistance is a condition wherein the pancreas must secrete an elevated amount of insulin compared to the usual levels required to maintain normal blood glucose levels. This condition arises due to reduced sensitivity or responsiveness of tissues to the biological activity of insulin. Globally, the prevalence of insulin resistance among adults varies, ranging from 15.5% to 46.5% [1]. Insulin resistance typically affects approximately 25% to 35% of the Western population and is frequently linked to obesity and its associated complications. These complications include type 2 diabetes mellitus (T2DM), cardiovascular disease, specific types of cancer, infertility, non-alcoholic fatty liver disease, and cognitive impairment [2].

In Oman, a study conducted among medical students revealed a notably high prevalence of insulin resistance, reaching 16% [3]. Furthermore, in Saudi Arabia, there is a growing incidence of insulin resistance-related disorders, as indicated by a recent survey that reported a diabetes mellitus prevalence of 23.7% among adults [4]. Globally, insulin resistance is approaching epidemic proportions, driven by sedentary lifestyles and diets rich in carbohydrates and trans-fats [5]. Several studies have also identified sleep patterns and sleep deprivation, along with chronic stress, as contributing factors to insulin resistance [6].

Insulin resistance is known to increase in tandem with rising body mass index (BMI), waist circumference, and particularly, waist-hip ratio. These parameters are indicative of increased adiposity, notably elevated levels of visceral adipose tissue. It has been observed that android-type obesity strongly contributes to the development of insulin resistance and type 2 diabetes. This type of obesity often coincides with conditions such as hypertension and dyslipidemia, characterized by high levels of triglycerides and low-density lipoproteins, as well as low levels of high-density lipoproteins [7]. Substantial evidence supports the therapeutic role of physical activity in managing insulin resistance and type 2 diabetes. A substantial body of research underscores the positive impact of exercise on improving insulin sensitivity and its beneficial effects in insulin-resistant conditions [8].

Stress is a state characterized by mental or emotional strain when an individual perceives environmental demands as surpassing their adaptive capacity. Stress can influence insulin resistance by triggering the hypothalamic-pituitary-adrenal (HPA) axis, leading to increased cortisol levels, which, in turn, contribute to elevated insulin resistance [9].

Additionally, sleep and sleep deprivation have been shown to raise fasting blood glucose levels in healthy young adults, along with alterations in diurnal cortisol secretion and reduced heart rate variability. There is also a growing body of evidence suggesting that chronic sleep deprivation can impact insulin levels and contribute to insulin resistance [10]. Moreover, it plays a pivotal role in the pathogenesis of various diseases, including T2DM, hypertension, dyslipidemia, and cardiovascular disorders [11].

The percentage of the prevalence rate serves as an indicator of the severity of the insulin resistance phase, which is regarded as a precursor to the development of complex diseases. Importantly, insulin resistance is relatively easy to prevent, underscoring the need to prioritize raising awareness about its early, less evident causes rather than solely focusing on addressing its consequences.

While there has been a limited amount of research conducted in Saudi Arabia to ascertain the prevalence of the mentioned risk factors, the majority of studies have examined the subsequent diseases stemming from insulin resistance in isolation, rather than exploring their deep-seated connections to insulin resistance itself.

General objective

The study’s overarching goal is to adopt the objectives outlined in a prior study conducted by Badawy et al. [12], one of the authors of this study. These objectives will be applied to male students from Ibn Sina College to investigate the factors that contribute to the onset of insulin resistance among them.

Specific objectives

This study aimed to analyze the lifestyle patterns of male students, measure blood pressure and postprandial glucose levels in male students, and evaluate anthropometric measurements among male medical students.

## Materials and methods

Study design and setting

This study replicates the study design and setting of research conducted by Badawy et al. [12] on female medical students from Ibn Sina College. In contrast, our study focuses on male medical students from the same college, with the aim of investigating the prevalence of contributing factors leading to the development of insulin resistance. Our research was performed at a private college of medical sciences in Saudi Arabia.

The study population consisted of male medical students enrolled in the medicine program across all academic year levels, ranging from year one to year six. Data collection occurred between January and December 2022, with the primary data collection period occurring from February to May 2022. Inclusion criteria for participation in the study required enrollment in a private medical college, while exclusion criteria included failure to complete the study measurements.

Sampling technique

A convenient non-probability sampling technique was employed for this study. The sample size was determined using the single population formula with Epi-info version 7. Based on an estimated prevalence of 23%, which was derived from a prior study in a similar cohort, and allowing for a margin of error of 5%, the calculated sample size was 196 [13].

Data collection

Each participant underwent an in-person interview, during which anthropometric measurements and biochemical testing such as postprandial blood sugar were conducted. The in-person interview involved a series of questionnaires designed to gather demographic data and assess lifestyle habits related to insulin resistance.

To assess lifestyle factors, validated questionnaires were employed. The International Physical Activity Questionnaire (IPAQ)-Short Form was utilized to gauge physical activity [14], while the Single-Item Sleep Quality Scale (SQS) was employed to evaluate sleep patterns [15]. To assess dietary patterns, the Healthy Eating Quiz (HEQ) was administered to rate participants’ adherence to healthy eating habits and identify areas for improvement in their diet [16]. Additionally, the stress questionnaire developed by the International Stress Management Association (ISMA) was used to assess stress levels [17].

Anthropometric data, including height (using a stadiometer), weight (using a digital scale), and waist circumference (using a tape and taking the umbilicus and just above the iliac crest as a landmark), were collected as part of the study. The cutoff points for high waist circumference were 102 cm or more, and for BMI, the cutoff points were 25-29.99 considered overweight but 30 or more considered obese. Biochemical testing involved the estimation of postprandial blood glucose levels using a digital glucometer, which was conducted on campus. The cutoff points for blood sugar were 140-199 mg/dL for prediabetics and 200 mg/dL or more for the diagnosis of diabetes. Furthermore, systolic and diastolic blood pressure measurements were taken using a mercury sphygmomanometer. The cutoff for elevated or high blood pressure was 120/80 mmHg or more.

Statistical analysis

Data were collected and organized using Microsoft Excel (Microsoft Corp., Redmond, WA, USA), and subsequent statistical analyses were conducted using SPSS statistics software (IBM Corp., Armonk, NY, USA). To calculate prevalence, percentages were employed. The chi-square test was utilized for the analysis of qualitative variables. The significance level chosen for this study was set at p-values <0.05.

Ethical considerations

Approval for this study was obtained from the Ethical Committee of the Ibn Sina National College for Medical Studies (approval number: IRRB-03-28022022). Strict confidentiality of all gathered information was maintained throughout the study. A consent form for participation was included in the data collection sheet.

## Results

Table 1 presents the sociodemographic characteristics and health status of undergraduate male medical students at a private college in Saudi Arabia. A total of 241 medical students participated in the study. Among the respondents, less than half (41.9%) were in the age range of 20-22 years, and more than one-third (37.3%) were in the age group of 23-24. In terms of education level, just under a quarter (22.8%) were in their first year, while around a quarter (20.3%) were in their fourth year, making them the highest participating groups.

**Table 1:** Sociodemographic and health status data among male medical students at a private medical college in Saudi Arabia (n = 241). ASCVD: atherosclerotic cardiovascular disease; CVD: cardiovascular disease

Variable		Frequency	Percentage
Age group	Less than 20	29	12.0
	20–22	101	41.9
	23–24	90	37.3
	25–28	21	8.7
Level of education	First	55	22.8
	Second	43	17.8
	Third	36	14.9
	Fourth	49	20.3
	Fifth	31	12.9
	Sixth	27	11.2
Smoking	Never	141	58.5
	Former	39	16.2
	Current	61	25.3
Having dyslipidemia	Yes	7	2.9
	NO	234	97.1
Having diabetes	Yes	10	4.1
	No	231	95.9
Having hypertension	Yes	6	2.5
	No	235	97.5
Having ASCVD	Yes	5	2.1
	No	236	97.9
Hormonal disorders	Yes	6	2.5
	No	235	97.5
Family history of dyslipidemia	Yes	42	17.4
	No	199	82.6
Family history of diabetes	Yes	82	34.0
	No	159	66.0
Family history of hypertension	Yes	88	36.5
	No	153	63.5
Family history of CVD	Yes	29	12.0
	No	212	88.0
On corticosteroids	Yes	5	2.1
	No	236	97.9

Regarding smoking status, more than half (58.5%) reported never smoking, while almost one-quarter (25.5%) were current smokers. Regarding the history of chronic illness, diabetes affected 4.1% of students, followed by dyslipidemia, which was reported by 2.9%. When students were interviewed about their family history of chronic diseases, hypertension was revealed to be present in 36.5% of families, and more than one-third (34.0%) reported a family history of diabetes. Similarly, 17.4% of students had a family history of dyslipidemia.

Table 2 shows the prevalence of risk factors known to be associated with insulin resistance among male medical students at a private medical college in Saudi Arabia. Regarding BMI, more than half (52.2%) of the students were overweight or obese. On the other hand, almost one-fifth (19.5%) of the students had a high waist circumference. Nevertheless, a small proportion (2.9%) had high blood sugar. Considering the systolic and diastolic blood pressure, 77.6% and 64.3% had elevated systolic and diastolic blood pressure, respectively. Less than one-third (29.5%) of the students suffered from stress. However, 56.8% of the students had poor night sleep. Regarding the diet, 39% of the students had an unhealthy diet. Moreover, the majority (59.8%) of the students had low physical activity.

**Table 2:** Prevalence of risk factors known to be associated with insulin resistance among male medical students at a private medical college in Saudi Arabia (n = 241).

Variable		Frequency	Percentage
Body mass index	Normal weight	114	47.3
	Overweight or obese	127	52.7
Waist circumference	Normal waist circumference	194	80.5
	High waist circumference	47	19.5
Blood sugar	Normal blood sugar	234	97.1
	High blood sugar	7	2.9
Systolic blood pressure	Normal	54	22.4
	Elevated or high	187	77.6
Diastolic blood pressure	Normal	86	35.7
	Elevated or high	155	64.3
Stress score	Less likely to suffer from stress	170	70.5
	More likely to suffer from stress	71	29.5
Night sleep score	Good night sleep	104	43.2
	Poor night sleep	137	56.8
Diet	Healthy diet	145	60.2
	Unhealthy diet	96	39.8
Physical activity	Low	144	59.8
	Moderate	48	19.9
	High	49	20.3
	Total	241	100

Table 3 shows the lifestyle risk indicators and their relation to waist circumference as an indicator of insulin resistance using the chi-square test. More than one-third (38.3%) of those with high waist circumference were more prone to stress, but it was not statistically significant when compared to those with normal waist circumference (p = 0.139). Moreover, more than two-thirds (68.09%) of those with high waist circumference showed poor sleep patterns at night, which was not statistically significant (p = 0.083). Unhealthy diet was more associated with 61.70% of high waist circumference participants, and it was statistically significant (p = 0.001). The majority (82.98%) of male medical students with high waist circumference who participated in this study were physically inactive compared to those with normal waist circumference (p = 0.001), which was highly statistically significant.

**Table 3:** Relation of lifestyle indicators and waist circumference as an indicator of insulin resistance among male medical students at a private college in Saudi Arabia. ^a^: based-on chi-square p-values; *: significant p-values.

Risk factors	High waist circumference (n = 47)	Normal waist circumference (n = 194)	P-value^a^
Less likely to suffer from stress	29 (61.70%)	141 (72.68%)	0.139
Prone to stress	18 (38.30%)	53 (27.32%)	
Good sleep	15 (31.92%)	89 (45.88%)	0.083
Poor sleep	32 (68.09%)	105 (54.12%)	
Healthy diet	18 (38.30%)	127 (65.46%)	0.001*
Unhealthy diet	29 (61.70%)	67 (34.54%)	
Low physical activity	39 (82.98%)	105 (54.12%)	0.001*
Moderate physical activity	4 (8.51%)	44 (22.68%)	
High physical activity	4 (8.51%)	45 (23.20%)	

**Figure 1:**
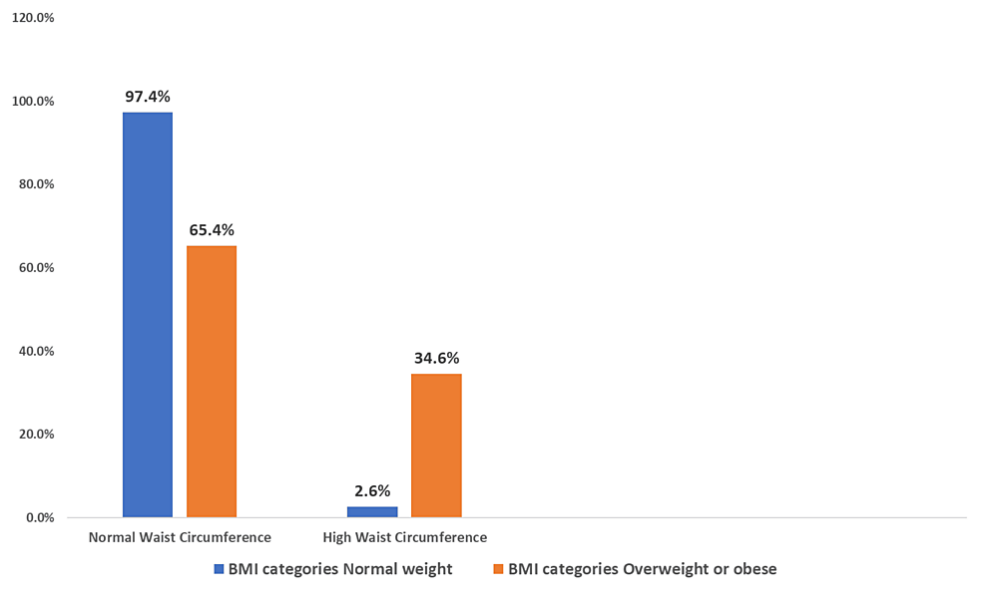
Clustered bar chart showing the association of waist circumference and body mass index (BMI) among male medical students at a private college in Saudi Arabia. Based on chi-square p-values of 0.000 which is highly statistically significant.

**Figure 2:**
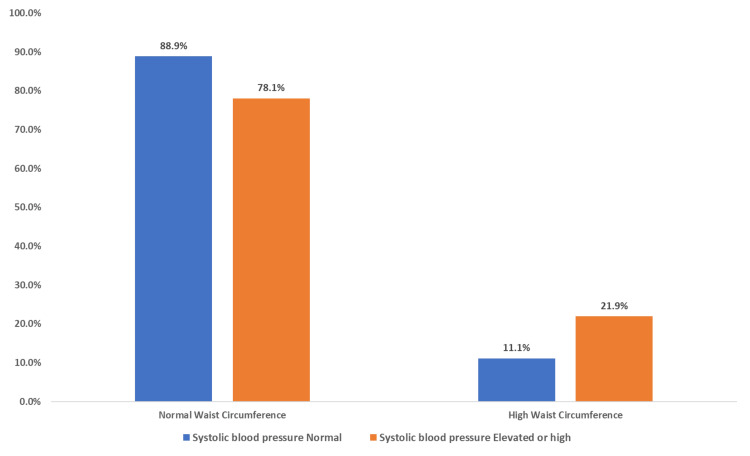
Clustered bar chart showing the association of waist circumference and systolic blood pressure among male medical students at a private college in Saudi Arabia. Based on chi-square p-values of 0.077 which is not statistically significant.

**Figure 3:**
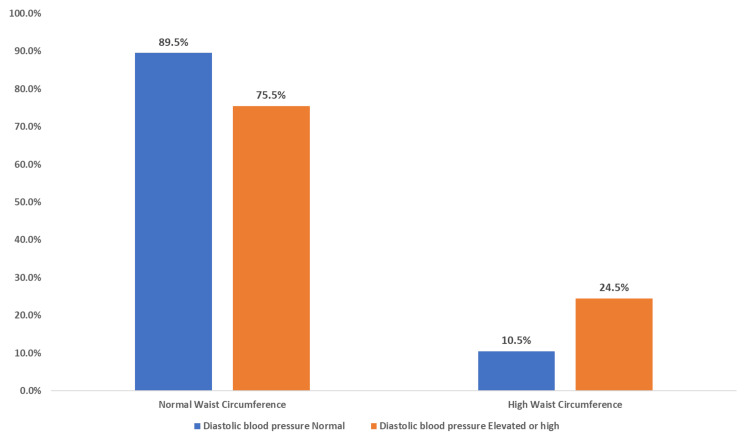
Clustered bar chart showing the association of waist circumference and diastolic blood pressure among male medical students at a private college in Saudi Arabia. Based on chi-square p-values of 0.008 which is statistically significant.

**Figure 4:**
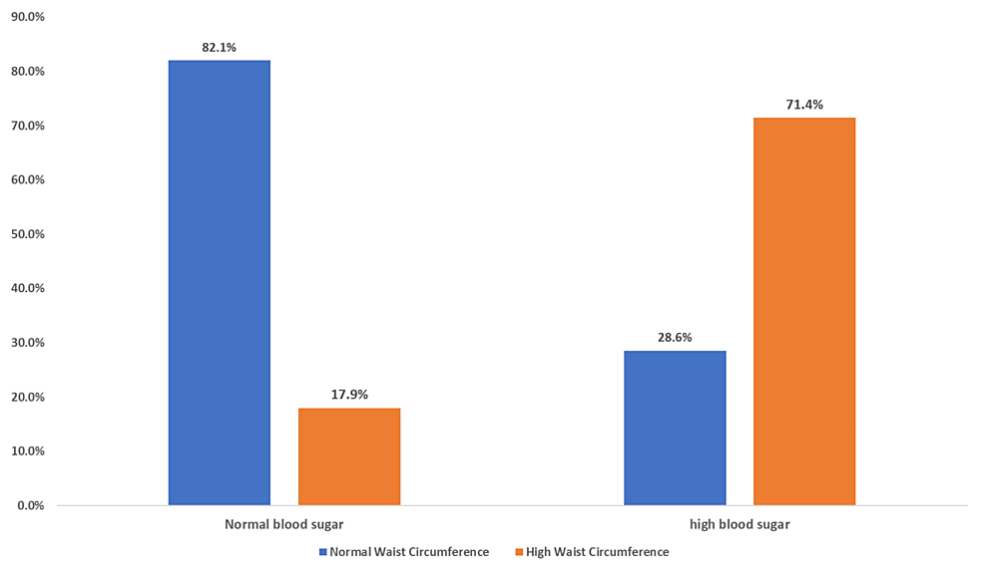
Clustered bar chart showing the association of waist circumference and blood sugar among male medical students at a private college in Saudi Arabia. Based on chi-square p-values of 0.000 which is highly statistically significant.

The clustered bar chart (Figure 1) illustrates the relationship between waist circumference as an indicator of insulin resistance and BMI. Among all participants in the study, less than one-fifth (18.26%) had both a high waist circumference and were overweight or obese, while a larger proportion (34.44%) had a normal waist circumference but were overweight or obese and 2.6% had a high waist circumference but their BMI was normal. This association, analyzed using the chi-square test, was found to be statistically significant (p < 0.001). When the relationship of waist circumference to systolic blood pressure was tested (Figure 2), no significant difference was detected (p = 0.077). A significant relationship was detected when testing the association between waist circumference and diastolic blood pressure (p = 0.008) (Figure 3). Additionally, we detected a significant relationship between waist circumference and blood sugar level (p = 0.000) (Figure 4).

## Discussion

The primary objective of this study was to assess the prevalence of contributing factors that lead to the development of insulin resistance among male medical students at a private college in Saudi Arabia. The strength of our study lies in its substantial sample size of young adults and its ability to explore a broad range of risk factors for insulin resistance, including lifestyle choices and selected personal and family history factors. The study revealed a high prevalence of several factors that play a significant role in the development of insulin resistance. First, in terms of smoking, nearly one-quarter of the male medical students in the present study were current smokers, which is a known risk factor for insulin resistance. A recent study also found that individuals who smoked for an extended period had a higher risk of developing insulin resistance [18].

Regarding a family history of hypertension and diabetes, which are strongly linked to insulin resistance, our study showed that more than one-third of participants had a family history of both conditions. Research has indicated that a family history of diabetes can influence insulin resistance and insulin secretion, as observed in the Chinese population [19]. Parental history of hypertension and type 2 diabetes, conditions associated with dyslipidemia, chronic hypertension, and obesity in men, has long been recognized as significant risk factors for metabolic syndrome [20].

The present study indicates that over half of the students had a BMI placing them in the overweight or obese category. These findings align with previous research, which identified factors such as insufficient physical activity, suboptimal dietary habits, excessive fructose consumption, and smoking as the predominant contributors to insulin resistance [21].

Conversely, when waist circumference measurements were taken, nearly one-fifth of the students exhibited an elevated waist circumference. This observation could potentially be attributed to the possibility that the higher body weight among the male students may primarily stem from increased muscle mass rather than a significant accumulation of body fat.

Only a small number of the students exhibited prediabetic or diabetic levels of blood sugar. These results contrast with those of a study conducted in India, which reported that nearly one-third of young medical students had moderate to high-risk diabetes scores [22].

Conversely, a significant majority of male medical students demonstrated elevated systolic or diastolic blood pressure. This finding may be attributed to the relatively low cutoff points for defining elevated blood pressure, with a normal systolic reading considered less than 120 mmHg and diastolic less than 80 mmHg.

A substantial proportion (59.8%) of participants in the current study reported low levels of physical activity. This trend can be attributed to the demanding schedules of medical students, leaving them with limited opportunities for physical activity.

Furthermore, more than half (56.8%) of the respondents reported experiencing nighttime sleep difficulties. These findings align with those of a cross-sectional study involving Saudi students, where one-third (33.8%) reported having a short sleep duration of less than seven hours per night [23].

The present study found that fewer than one-third of male medical students reported experiencing stress. These results are consistent with a study conducted among Thai medical students, which found that approximately 61.4% of students experienced some level of stress [24].

According to the findings of this study, 39.8% of respondents reported having an unhealthy diet. These results are consistent with a study conducted in Saudi Arabia, as mentioned by Al-Qahtani, which noted that “contrary to the expectations and regardless of studying in medical college, our medical students; both male and females at different academic levels are having major bad dietary habits and life style that is comparable to the general population in the kingdom.”

The study assessed the association between lifestyle risk indicators and their correlation with waist circumference as an indicator of insulin resistance, specifically focusing on stress and nighttime sleep. The findings revealed that more than one-third of students with a high waist circumference experienced stress, while more than two-thirds of those with a high waist circumference reported poor sleep. Surprisingly, both variables did not exhibit statistically significant differences when compared to those with a normal waist circumference. However, it is worth noting that a study involving Chinese individuals discovered an inverse correlation between perceived stress and obesity [26]. Additionally, various studies have highlighted a substantial association between sleep patterns and waist circumference [27].

An unhealthy diet exhibited a stronger association with participants having a high waist circumference, and this association was statistically significant when compared to those with a normal waist circumference. These findings were supported by a recent study in Iran that reported a significant and negative relationship between a healthy dietary pattern and insulin resistance [28].

In the realm of physical activity, it was observed that a significant number of male medical students with elevated waist circumference, who participated in this study, exhibited a lack of physical activity when compared to their counterparts with a normal waist circumference. This discrepancy is highly statistically significant. These findings align with numerous other studies that have highlighted a strong association between central obesity and low levels of physical activity [29].

A study conducted in the Kingdom of Saudi Arabia has confirmed the increasing prevalence of disorders related to insulin resistance, which has reached 23.7% in adults [4]. The data collected in this study primarily examined the relationship between waist circumference, as an indicator of insulin resistance, and variables such as BMI, systolic blood pressure, diastolic blood pressure, and blood glucose levels. The current findings show a statistically significant association between BMI and higher waist circumference as a marker of insulin resistance. An earlier study supported this link, particularly emphasizing that android-type obesity is a significant contributor to the development of insulin resistance and type 2 diabetes [7].

Regarding systolic blood pressure, unexpectedly, the present study did not find a significant difference between individuals with high and low waist circumferences. However, a notable relationship was observed for diastolic blood pressure. These results are in line with earlier studies on the relationship between waist circumference and blood pressure. It was hypothesized in these studies that there is a well-established association between insulin sensitivity and blood pressure, with fasting insulin possibly serving as a crude marker for the metabolic abnormalities associated with insulin resistance [30].

Blood sugar levels were found to have a statistically significant association with waist circumference, which aligns with the findings of numerous studies. Recent research has underscored a robust connection between an enlarged waist circumference and an elevated risk of developing type 2 diabetes, irrespective of an individual’s BMI. These studies have shown that individuals who are overweight and have a large waist circumference (defined as exceeding 40 inches/102 cm for men and 34.5 inches/88 cm for women in these studies) face a similar risk of diabetes development as those who are clinically obese [5].

Recommendation

Lifestyle modifications, such as improving one’s diet and increasing physical activity, serve as the primary methods for preventing insulin resistance. The data presented in this study underscore the importance of public education and raising awareness about these issues.

Study limitations

This study has several limitations worth noting. First, it was conducted exclusively among young male medical students, making it not fully representative of the broader population. The cohort’s risk factors may differ from those of the general Saudi population, and the study’s narrow age range and absence of female participants further limit its generalizability. Not all criteria for insulin resistance diagnosis were included. The study is, therefore, susceptible to selection bias.

Additionally, different examiners were involved in taking anthropometric measurements, introducing the possibility of interexaminer variability. Lastly, data collected through self-reported questionnaires on physical activity, stress, night sleep, and diet may be subject to participant subjectivity. This subjectivity can lead to potential under-reporting or over-reporting of certain factors.

## Conclusions

This study highlighted that increased waist circumference, as an indicator of insulin resistance, was notably high among medical students. Furthermore, many well-known risk factors contributing to insulin resistance were significantly elevated in this group of students. The prevalence of overweight/obesity was also notable, with a direct correlation between BMI and insulin resistance. Therefore, promoting a healthy diet and physical activity is imperative in this context.
